# Pulmonary effects of passive smoking: the Indian experience

**DOI:** 10.1186/1617-9625-1-2-129

**Published:** 2002-06-15

**Authors:** D Gupta, AN Aggarwal, SK Jindal

**Affiliations:** 1 Department of Pulmonary Medicine, Postgraduate Institute of Medical Education and Research, Chandigarh, India

## Abstract

There are only a few studies done on pulmonary effects of passive smoking from India, which are summarized in this paper. Several vernacular tobacco products are used in India, bidis (beedis) being the commonest form of these. Bidis contain a higher concentration of nicotine and other tobacco alkaloids compared to the standard cigarettes (e.g., the sum of total nicotine and minor tobacco alkaloids was 37.5 mg in bidi compared to 14–16 mg in Indian or American cigarettes in one study). A large study performed on 9090 adolescent school children demonstrated environmental tobacco smoke (ETS) exposure to be associated with an increased risk of asthma. The odds ratio for being asthmatic in ETS-exposed as compared to ETS-unexposed children was 1.78 (95% CI: 1.33–2.31). Nearly one third of the children in this study reported non-specific respiratory symptoms and the ETS exposure was found to be positively associated with the prevalence of each symptom. Passive smoking was also shown to increase morbidity and to worsen the control of asthma among adults. Another study demonstrated exposure to ETS was a significant trigger for acute exacerbation of asthma. Increased bronchial hyper-responsiveness was also demonstrated among the healthy nonsmoking adult women exposed to ETS. Passive smoking leads to subtle changes in airflow mechanics. In a study among 50 healthy nonsmoking women passively exposed to tobacco smoke and matched for age with 50 unexposed women, forced expiratory volume in first second (FEV1) and peak expiratory flow (PEF) were marginally lower among the passive smokers (mean difference 0.13 L and 0.20 L-1, respectively), but maximal mid expiratory flow (FEF25–75%), airway resistance (Raw) and specific conductance (sGaw) were significantly impaired. An association between passive smoking and lung cancer has also been described. In a study conducted in association with the International Agency for Research on Cancer, the exposure to ETS during childhood was strongly associated with an enhanced incidence of lung cancer (OR = 3.9, 95% CI 1.9–8.2). In conclusions several adverse pulmonary effects of passive smoking, similar to those described from the western and developed countries, have been described from India.

## Introduction

Passive smoking or environmental tobacco smoke (ETS) exposure has been variously described as 'second-hand smoke' or 'involuntary smoking'. In the past, little attention, beyond its nuisance effect, was paid to the health consequences of passive smoking. Exhaustive report on health consequences of involuntary smoking by the United States Surgeon General [1] and reports by the United States Environmental Protection Agency [2] highlighted the increased risks of several diseases similar to those seen among smokers, in persons exposed to ETS at home or at work place. Several hundred studies were quoted in these reports, which cited various pulmonary and extra-pulmonary health effects of passive smoking. But most of these studies were reported from the Western and developed countries. There are very few reports on the health effects of passive smoking from the developing and the under-developed countries.

Smoking is on the rise in developing countries although there are differences in tobacco products, quality of tobacco used and the smoking practices [3]. Environmental conditions such as the overcrowding and the poor ventilation at homes and work places may make the health effects of ETS more pronounced. There are a few studies on pulmonary effects of passive smoking from India. In this paper, we review the published information available from India on pulmonary effects of passive smoking.

## Composition of ETS

Tobacco smoke contains over 4000 chemicals in the forms of particles and gases [2]. ETS is a combination of sidestream (SS) smoke emitted from the burning end of a cigarette and the remainder of mainstream (MS) smoke exhaled by a smoker. The sidestream smoke constitutes about 85% of the smoke present in the room where active smokers smoke, and contains many potentially toxic components, some of which may occur in even higher concentrations than in the mainstream smoke [4]. The particulate phase consists of tar (itself composed of many chemicals), nicotine, benzo(*a*)pyrene, and hundreds of other noxious compounds. A few examples of gases in tobacco smoke are carbon monoxide, benzene, ammonia, dimethylnitrosamine, formaldehyde, hydrogen cyanide and acrolein. Some of these constituents have marked irritant properties and there are found to be about 60 known or suspected human carcinogens present in tobacco smoke. The environmental tobacco smoke has been classified as class A (known for human) carcinogen along with asbestos, arsenic, benzene and radon gas [5].

The most popular Indian smoking product is "bidi" (also spelled as beedi). Bidis are made of crude sun-dried tobacco wrapped in a dried Tendu (Dyospyros melanoxylon) leaf. Another smoking product used in different parts of India is "chillum" or "hooka", which resembles a pipe made of clay. Tobacco is burnt along with molasses and coal, and smoked either directly or through a long pipe with smoke passing through a water container. The amount of nicotine and tobacco alkaloids present in the mainstream smoke (MS) of these vernacular tobacco products is likely to be different from those present in the MS of standard cigarettes because of the differences in their design (e.g. water acts as a filter in hooka and no filter exists in most bidis). The sidestream (SS) smoke released from such products is also likely to be different from the SS of standard cigarettes due to differences in tobacco processing, burning rate and temperature, and the use of additives for burning tobacco. In a study from Mumbai, the bidi, an Indian cigarette and a brand of American cigarette were analyzed by gas chromatography-flame ionization detection for the levels of nicotine and minor tobacco alkaloids in the MS and SS smoke [6]. The analysis demonstrated higher total nicotine and minor tobacco alkaloids in tobacco from bidi (37.7 mg/g) compared to Indian or American cigarettes (14–16 mg/g). This study also demonstrated higher delivery of nicotine and alkaloids by bidi as evidenced by higher concentration of nicotine in the MS smoke (MS/SS) compared to that released by a regular cigarette. In another study from Mumbai, air samples from different indoor environments were analyzed for the levels of various volatile organic compounds. A very high level of benzene was detected in a smoker's room and in a kitchen using kerosene as fuel [7].

## Quantifying Passive Smoking

One of the very difficult tasks in most studies on passive smoking is to quantify an ETS exposure. Most epidemiological studies of ETS depend largely on the validity of self-reported exposure. In a large multi-country collaborative study, which included Chandigarh as one of the collaborating centers, the self-reported ETS exposure among nonsmoking women married to smokers at home or at work was evaluated. This study was conducted in 13 centers across 10 countries and included 1,369 nonsmoking married women. Urinary cotinine levels were measured and correlated with the history of ETS exposure. It was demonstrated that nonsmoking women could provide appropriate estimates of their exposure, which well correlated with their biochemically measured exposure levels. The results of a linear regression analysis indicated that the duration of exposure and the number of cigarettes to which the subjects reported to be exposed were strongly correlated with the levels of urinary cotinine excretion. The number of cigarettes was the best measure of exposure to husbands' smoking, while exposure at workplace was more strongly related to the duration of exposure [8]. It was also shown that the potential bias due to smoker misclassification was very unlikely to be responsible for the increased health risks observed in the epidemiological studies on ETS [9].

## Passive Smoking and Lung Functions

There is a biological plausibility that passive smoking can affect the pulmonary functions similar to the effects of active smoking. There are only a few cross-sectional and longitudinal studies that have shown ETS exposure as an important risk factor for obstructive lung disease with a significant dose response relationship [10,11]. Controlled acute exposure to ETS also results in some decrements in lung volumes and flows [12].

In a study from Chandigarh on 200 school children, the effects of passive smoking and domestic cooking fuels on lung function were evaluated. It was observed that spirometric indices were lower in children exposed to biomass fuels vis-a-vis liquid petroleum gas at home. All the indices studied such as vital capacity (VC), forced expiratory volume in first second (FEV_1_), FEV_1_/VC ratio, peak expiratory flow (PEF) and maximal mid expiratory flow (FEF_25–75%_) were also lower among passive smokers, though statistically significant difference was observed only in mid expiratory flows among children exposed to mixed fuels [13].

Recently, we have also studied airflow mechanics in asymptomatic healthy women to evaluate the effects of long-term exposure to ETS (unpublished data). Lung functions [including VC, FEV_1_, FEV_1_/VC ratio, PEF, FEF_25–75%_, airway resistance (R_aw_) and specific conductance (sG_aw_)] were compared among 50 healthy nonsmoking women exposed to passive smoking (Group I) and age matched 50 women not similarly exposed (Group II). Conditional and logistic linear regression analyses were performed to assess contribution of household ETS-exposure to decreased lung function after adjusting for potential confounders. The results have demonstrated that although FEV_1 _and PEF of passive smokers were only marginally lower than the controls (mean difference 0.13 L and 0.20 L^-1^respectively), their FEF_25–75%_, R_aw _and sG_aw _were significantly impaired. Ten (20%) women exposed to ETS and five (10%) unexposed had abnormal R_aw _(adjusted odds ratio 6.72, 95% confidence interval 1.15–39.42), while eight (16.0%) women in group I and only 1 (2%) in group II had abnormal sG_aw _(adjusted odds ratio 21.08, 95% CI = 1.30–341.05).

Bronchial hyper responsiveness (BHR) is an important determinant of decline in lung function in normal subjects and those with chronic bronchitis [14]. Parental smoking has shown to be associated with BHR in children [15]. We have studied BHR, as measured by methacholine broncho-provocation test, in three groups of nonsmoking housewives with or without history of exposure to ETS or biomass fuel combustion [16]. The first group comprised of 60 controls, all being nonsmokers with no history of chronic exposure to environmental tobacco smoke (ETS) or biomass fuels. Three of these women showed a 20 per cent FEV_1 _fall with a cumulative methacholine dose of 72.5 mg or less. Of 60 women in group II (ETS-exposed) and 52 in group III (biomass fuel exposed), 26 (43.3%) and 10 (19.2%) showed bronchial BHR, respectively. The odds ratios for BHR in groups II and III were 14.53 and 4.52, respectively, compared to controls. The number of hyper-responders was significantly more and the mean PD_20 _was less among the exposed than among the unexposed group. The occurrence of BHR was more evident among the ETS-exposed group (P < 0.05) than among the biomass-combustion group. There were more hyper-responders among group II and those who had an overall higher cumulative exposure as measured by an exposure index of 50 or more [exposure index (EI) is equal to an average daily number of cigarettes or bidis to which exposure is reported, multiplied by the number of years during which such an exposure occurred], compared to those with EI of less than 50. An ETS-exposure may thus either unmask the inherent BHR or sensitize hitherto non-reactive airways.

Passive smoking can cause subtle changes in lung function. Several earlier studies have also shown similar findings. The magnitude of reported changes, however, has been small both in cross-sectional and longitudinal studies. Some investigators have reported a significant decrease in FEV_1 _[17,18], while some others have documented non-significant lower values [19,20]. A significant decrement in the mid-expiratory flow rates observed in our studies on both children and adult women, points towards narrowing of the small airways in a fashion similar to the known observations among active smokers [21]. However, larger longitudinal trials are needed to evaluate progression of this impairment with continued exposure to ETS.

## Passive Smoking and Asthma

The noxious effects of passive smoking on asthma are frequently described and debated. Passive smoking has been linked to the causation, increased morbidity and acute exacerbation of asthma [22].

The role of ETS in causing asthma has now been worldwide accepted. The International Consultation on Environmental Tobacco Smoke (ETS) and Child Health held in Geneva, Switzerland, concluded that ETS exposure causes a wide variety of adverse health effects in children, including lower respiratory tract infections such as pneumonia and bronchitis, coughing and wheezing, initiation and worsening of asthma, and middle ear disease [23]. Cross sectional studies have consistently revealed a detrimental effect of parental smoking, especially maternal, on lung function and/or severity of asthma in children [24]. There are increases in both prevalence and severity of asthma in children exposed to ETS from parental smoking. We have conducted a large community based survey for prevalence of asthma among adolescent school children in Chandigarh [25]. Using a previously standardized questionnaire [26], data from 9090 students in the 9 to 20 year age range were analyzed. There were 4367 (48%) boys, among whom the observed prevalence of asthma was 2.6%. Among 4723 (52%) girls, asthma was present in 90 (1.9%) students. A greater number of asthmatic students had either smoking parents or other family members who smoked at home as compared to non-asthmatics (41% vs. 28%, p < 0.0001). The odds ratio for being asthmatic for patients exposed to ETS compared to unexposed patients was 1.78 (95% confidence interval 1.33–2.31). Another study from the neighbouring state of Haryana, involved 2000 school children in rural settings using the International Study of Asthma and Allergy in Children (ISAAC) questionnaire [27]. Among these, forty children were found to be asthmatic. Each asthmatic child was matched with two healthy kids of the same age and sex from the same study population. An in-depth interview on possible risk factors was done for each case and its controls. The results of the multivariable analyses have shown that passive smoking was an important risk factor associated with asthma (OR 3.33, 95% CI = 1.85–7.65), besides the other factors such as having pets at home and the absence of windows in living rooms [28]. However, a smaller hospital based study in children from Delhi, failed to show any significant risk of developing asthma with passive smoking [29]. Conceptually, children, whose respiratory and immune systems are in the developing state, are more vulnerable than adults to ETS exposure [30]. Children also spend more time at home and get exposed to smoking from parents. In a meta-analysis, the pooled odd's ratio for asthma prevalence from 14 case control studies was 1.37 (95% CI 1.15–1.64) [31]. The International Consultation report referring to this latter has also concluded that both asthma and respiratory symptoms (wheeze, cough, breathlessness and phlegm) are indeed increased among children whose parents smoke; the results of over 60 studies on school-aged children have shown that the pooled relative risks for either parent smoking range from 1.2 to 1.4 [23].

ETS has adverse effects on asthmatic adults too. Various observational studies have reported worsening or precipitation of respiratory and nasal symptoms, cough and wheeze on exposure to ETS in allergic individuals [32]. One of the earliest studies documenting adverse effects of passive smoking on asthma in adults was conducted at our center [33]. The study was undertaken to compare the indices of morbidity and control of asthma in 100 adult patients exposed to ETS (group 2), with 100 asthmatics not exposed (group 1). Exposure was established from the history of smoking by the patient's spouse and other close contacts. Indices of asthma control and morbidity included the emergency department (ED) visits, hospitalization, acute episodes, requirement of parenteral drugs at home, corticosteroids, and maintenance bronchodilators in the preceding 1-year period. An index per patient was also calculated. Lung function was recorded by the measurement of forced expiratory flows on the same day of the follow-up visit. The mean age and disease duration were comparable, but the expiratory flows were lower in the patients exposed to ETS. More patients in this group had required daily bronchodilators (66 percent) and intermittent corticosteroids (56 percent) than patients from the other group. The number of ED visits, acute episodes, and parenteral bronchodilators per patient were significantly more (p < 0.01) in group 2 patients. Similarly, the number of weeks of absence from work and of corticosteroids requirement were more (p < 0.01) among the ETS-exposed patients (Table 1). Subsequently, we had studied the role of ETS exposure in causing acute exacerbations of asthma [34]. One hundred patients of asthma seen in the emergency room with an acute attack of less than 24 hour duration were interviewed for exposure to known asthma triggers including ETS in the preceding 24 hours and were compared with 100 stable patients of asthma. Sixty-seven patients with acute exacerbation could point to a possible triggering factor. There was a significantly higher prevalence of exposure to ETS in patients with acute exacerbations compared to stable asthmatics (41% vs. 20%; p < 0.01). ETS was the only significant identifiable trigger for acute asthma in 33 ETS-exposed patients.

**Table 1:** Indices of asthma control (per patient in the preceding 1 year) in ETS exposed and unexposed asthmatic patients

	Group 1 (n = 100)	Group 2 (n = 100)
Emergency department visits	0.6	0.82*
Hospitalization	0.33	0.34
Acute episodes	0.6	1.32*
Parenteral bronchodilators (no.)	6.0	8.6*
Absence from work (weeks)	3.0	3.6*
Steroid requirement (weeks)	8.6	11.3*
Bronchodilators requirement (weeks)	36.3	38.3

* p < 0.01; Group 1 = Not exposed to ETS; Group 2 = Exposed to ETS (Reproduced with permission from reference 33).

Bronchial hyper-responsiveness is central to pathophysiology of asthma. We have also studied the effect of chronic ETS exposure on bronchial responsiveness by measuring BHR in stable, nonsmoking asthmatic women and compared the PD_20 _in ETS exposed and unexposed patients [35]. Histamine broncho provocation test was performed on 50 patients with stable asthma, of whom 23 (46%) were exposed to ETS (mean exposure 1.22 ± 0.61 hours/day for 13.07 ± 6.1 years). The PD_20 _was significantly lower in the ETS-exposed than in unexposed group (mean 5.66 ± 9.62 vs. 11.8 ± 13.06 units, median 1.7 vs. 6.1 units). The morbidity indices in the ETS group were also worse than in the control group. It is, therefore, likely that continued and chronic ETS exposure, especially when heavy, worsens asthma control by exacerbation of BHR. The adverse effects of ETS on asthma are corroborated by several inhalation challenge studies [36,37].

## Passive Smoking and Respiratory Symptoms in Children

Passive smoking increases the risk of lower respiratory tract infections such as bronchitis, pneumonia and bronchiolitis in children. The International Consultation report [23] concluded that parental smoking is an important cause of lower respiratory tract illnesses (e.g. croup, bronchitis, bronchiolitis, and pneumonia) during the first years of life. Of over 40 studies, all but one, have reported an increased risk among children whose parents smoke. Pooling the studies' results, children whose mothers smoke are estimated to have a 1.7-fold (95% CI = 1.6–1.9) higher risk of these illnesses than children of nonsmoking mothers. Paternal smoking alone causes a 1.3-fold (95% CI = 1.2–1.4) increase in risk. This result shows a strong evidence for a causal role of ETS exposure, since it is uncomplicated by maternal smoking during pregnancy. Similar effects were also seen for both wheezing and non-wheezing illnesses, and across studies done in communities and hospitals. In an asthma prevalence study we mentioned before [25], 31% of students reported the presence of one or more respiratory symptoms. ETS was also positively associated with prevalence of all respiratory symptoms, with odds ratios varying between 1.6 and 2.25 (Table 2). Similarly, a study was carried out to find out the prevalence and common causes of chronic/recurrent cough among rural children in Ludhiana (Punjab), India [38]. The prevalence of cough was 1.06% among a study sample of 2275 children. Asthma was the commonest cause of this cough (66.7%) followed by post-nasal drip (25%). Significantly, the family history of smoking was present in 16.7% of children with cough compared to 6.4% of children without cough.

**Table 2:** Prevalence of respiratory symptoms and asthma with reference to ETS exposure at home

	Exposed (n = 2574)	Unexposed (n = 6516)	Crude O.R. (95% C.I.) *	Age and sex adjusted O.R. (95% C.I.) *
Asthma (questionnaire diagnosis)	84 (3.3%)	121 (1.9%)	1.783 (1.345–2.364)	1.780 (1.340–2.364)
Symptoms				
Wheeze	226 (8.8%)	364 (5.6%)	1.627 (1.369–1.933)	1.605 (1.349–1.910)
Chest tightness	220 (8.5%)	345 (5.3%)	1.671 (1.402–1.992)	1.690 (1.417–2.017)
Dyspnea on exertion	425 (16.5%)	676 (10.4%)	1.708 (1.499–1.948)	1.689 (1.481–1.927)
Dyspnea at rest	129 (5.0%)	207 (3.2%)	1.608 (1.284–2.013)	1.671 (1.333–2.095)
Dyspnea at night	134 (5.2%)	205 (3.1%)	1.690 (1.353–2.112)	1.702 (1.360–2.129)
Cough at night	355 (13.8%)	556 (8.5%)	1.715 (1.488–1.976)	1.754 (1.521–2.023)
Cough in morning	210 (8.2%)	308 (4.7%)	1.790 (1.493–2.047)	1.763 (1.469–2.117)
Phlegm in morning	384 (14.9%)	586 (9.0%)	1.773 (1.545–2.035)	1.761 (1.533–2.023)
Phlegm for >3 months	152 (6.7%)	224 (4.0%)	1.698 (1.374–2.099)	1.719 (1.388–2.129)
Chest tightness on exposure to allergens	425 (16.5%)	526 (8.1%)	2.252 (1.964–2.583)	2.245 (1.956–2.577)
Dyspnea on exposure to allergens	432 (16.8%)	658 (10.1%)	1.796 (1.575–2.047)	1.881 (1.648–2.147)

* Odds ratio and 95% confidence interval (Reproduced with permission from reference 25).

## Passive Smoking and Lung Cancer

Nonsmoking individuals with long-term ETS-exposure have an increased risk of lung cancer. Hackshaw et al. reviewed 37 epidemiological studies on the risk of lung cancer in nonsmoking persons (4626 cases) [39]. It was found that lifetime risk of lung cancer in nonsmoking persons who lived with a smoker was 24% (95% CI 13% to 20%). Tobacco specific carcinogens have also been detected in the blood of the ETS exposed non-smoking persons providing clear evidence of the ETS association. A dose-response relationship between the nonsmoker's risk of lung cancer and cumulative exposure to ETS was also demonstrated in this review [39]. More recently, Taylor and colleagues have done a cumulative meta-analysis incorporating 43 studies (meeting their inclusion criteria from a total 76 primary epidemiological studies and 20 meta-analyses reported between 1981 and 1999) [40]. The pooled relative risk (RR) for never-smoking women exposed to ETS from spouses, compared with unexposed never-smoking women was 1.29 (95% CI 1.17–1.43).

**Figure 1:**
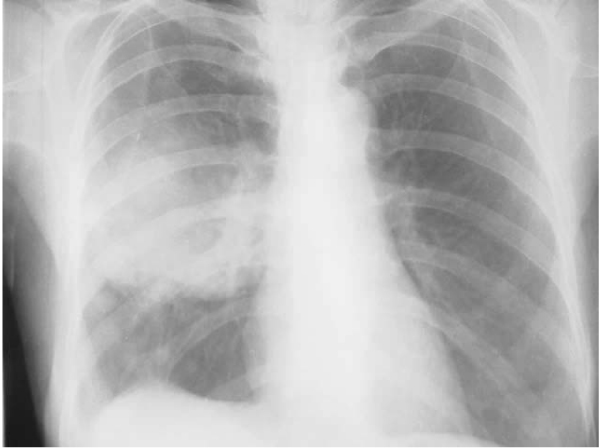
**A chest roentgenogram (PA view) of a non-smoking housewife with prolonged history of ETS exposure from father and spouse showing a mass lesion in right mid-zone, which turned out to be bronchogenic squamous cell carcinoma**.

In our experience, active smoking remains the strongest risk factor for developing lung cancer particularly among men. The association of lung cancer with smoking seems to be weaker in women as compared to men; an observation based on a study that only about one third of our female patients with lung cancer were smokers compared to nearly 90% of men with such malignancies [41]. In a recent study by us, we have interviewed 265 histologically confirmed lung cancer patients (235 men, 30 women) and 525 hospital controls (435 men, 90 women) matched for age and sex, by a pre-designed questionnaire. The effects of individual variables defining various aspects of tobacco smoking, indoor and outdoor air pollution and occupational exposure were assessed using unconditional logistic regression models. Eighty nine per cent of men and 33 per cent of women among the patients were ever-smokers as compared to 60 per cent of men and 20 per cent of women among the controls. The Odds Ratio (OR) for ever-smoking was 5.0 (CI 3.11–8.04) among men and 2.47 (CI 0.79–7.75) among women [42]. We have looked into the role of passive smoking in 58 histologically proven cases of nonsmokers' lung cancer using a case-control study with two age-and-sex matched controls per each patient [43]. Subjects were asked about their ETS exposure from different tobacco products beginning from childhood onwards at home, at workplace and in vehicles. Multivariable logistic analysis was done to assess the effects of ETS exposure on lung cancer. Exposure to ETS during the childhood was strongly associated with lung cancer (OR = 3.9, 95% CI 1.9–8.2) (Fig. 1). Restricting the analysis to women produced higher estimates of the risk (OR = 12, 95% CI 4.3–32). The observed risk was higher for ETS exposure through cigarettes as compared to *bidis *or *chilum*; a finding that is consistent with the observation of comparative composition of MS and SS smoke from different tobacco products as mentioned before [6]. A weaker association was seen between lung cancer and ETS exposure from spouse, at workplace and in vehicles. Another interesting observation was made in the earlier collaborative study of the International Agency for Research on Cancer (IARC) in Chandigarh as one of the study centers [44]. Some of the excess risk of lung cancer development in nonsmoking women from spousal smoking was attributable to the childhood ETS-exposure from parental smoking. It was also shown that indeed daughters of smoking parents more often tended to marry smokers (71% of women with smoking husbands had one or both parents smoking compared to 60.3% of women married to nonsmokers, who had at least one smoking parent; OR for the daughter of a smoker to marry a smoker was 1.64, 95% CI = 1.24–2.17), thus compounding the effects of parental and spousal ETS exposure. Therefore, ETS exposure, which is a recognized risk factor in countries with high prevalence of smoking, is also a risk factor in India, which historically has a low prevalence of smoking and lung cancer [45].

To summarize, passive smoking has several subtle as well as overt pulmonary effects. It is an established risk factor for lung cancer in nonsmoking persons. It is a significant risk factor for the respiratory symptoms and asthma in children. It is associated with an increased morbidity from asthma in adults, which is difficult to control. Passive smoking can also lead to a poor lung function, small airway dysfunction, and increased bronchial hyper-responsiveness in asymptomatic nonsmokers.

## Competing interests

The authors declare that they have no competing interests.
